# Prevalence and Characteristics of Dyslipidemia in a Hospital in Madagascar

**DOI:** 10.7759/cureus.73424

**Published:** 2024-11-11

**Authors:** Rova Malala Fandresena Randrianarisoa, Abderemane Abdoul-Kader, Mirantosoa Fabiola Ravelonjatovo, Narindrarimanana Avisoa Randriamihangy

**Affiliations:** 1 Department of Internal Medicine, University Hospital Joseph Raseta Befelatanana, Antananarivo, MDG; 2 Department of Cardiology, Mahavoky Atsimo University Hospital, Mahajanga, MDG

**Keywords:** cardiovascular risk factors, cholesterol, dyslipidemia, madagascar, prevalence

## Abstract

Introduction

Dyslipidemia is a major risk factor for atherosclerosis and is included in the metabolic syndrome. Data on dyslipidemia are still lacking in some parts of Africa. Our objectives were to report the prevalence of dyslipidemia and to describe the lipid profile of patients in a hospital in Madagascar.

Materials and methods

This was a descriptive cross-sectional study of patients admitted to the medical departments of Mahavoky Atsimo Hospital in Mahajanga, Madagascar, and followed for a period of 15 months. To be eligible, patients had to be over 18 years of age, have a lipid panel including total cholesterol, triglycerides, HDL, and LDL cholesterol, and agree to participate in the study.

Results

Of the 384 patients included in the study, 262 patients (68.23%) had dyslipidemia. The prevalence of dyslipidemia was 61.45% (n = 102) in men and 73.39% (n = 160) in women. In patients older than 65 years, the prevalence was 70.64% (n = 77). The different types of dyslipidemia were distributed as follows: hypercholesterolemia (24.22%, n = 93), hypo-HDL cholesterolemia (22.4%, n = 86), mixed hyperlipidemia (7.81%, n = 30), hyper-LDL cholesterolemia (7.29%, n = 28), and hypertriglyceridemia (6.51%, n = 25). Female gender (p = 0.013), diabetes mellitus (p = 0.007), and morbid obesity (p = 0.036) were associated with dyslipidemia.

Conclusion

The prevalence of dyslipidemia was high. Pure hypercholesterolemia and hypo-HDL-c were the most common types. Female gender, diabetes mellitus, and morbid obesity were associated with dyslipidemia. Prevention and treatment programs are essential to reduce the prevalence of dyslipidemia and the risk of cardiovascular events in low-income countries.

## Introduction

Cardiovascular disease (CVD) is a leading cause of mortality and morbidity worldwide [1]. The major risk factors are age, gender, dyslipidemia, hypertension, diabetes, obesity, and smoking. Dyslipidemia is a modifiable risk factor and a major component of metabolic syndrome [2]. It is characterized by high levels of total cholesterol, triglycerides, and low-density lipoprotein cholesterol (LDL-c) and/or low levels of high-density lipoprotein cholesterol (HDL-c) [3]. Dyslipidemia multiplies the risk of heart failure, ischemic heart disease, and stroke [4]. According to the World Health Organization (WHO), the global prevalence of hypercholesterolemia in adults was 39% in 2008 [5]. It is estimated that dyslipidemia is responsible for more than four million deaths per year [6]. In Africa, the prevalence ranges from 5.2% to 89.9%, with higher frequencies in East African regions [7,8].

Approximately 80% of the global burden of CVD disease occurs in low- and middle-income countries [9]. In Madagascar, CVD was responsible for nearly 20% of deaths, according to the 2018 WHO report [10]. Diabetes is common, with a national prevalence estimated at 3.9% in 2016 [11]. In the capital, Antananarivo, the prevalence of hypertension was 28.05% in 2009 [12]. Data on the characteristics of dyslipidemia are lacking. However, it is often associated with hypertension and diabetes. Assessing the prevalence of dyslipidemia can predict the course of CVD. Understanding the associated factors is essential to prevent cardiovascular events and reduce the socioeconomic burden of CVD [13]. Our objectives were to report the prevalence of dyslipidemia and to describe the lipid profile of patients.

## Materials and methods

Study characteristics

This was a descriptive cross-sectional study conducted at the medical departments of the Mahavoky Atsimo University Hospital in Mahajanga. Mahajanga is located in the northwest of Madagascar and is the capital of the Boeny region.

Study population and sampling

The study included patients who were admitted to the medical departments of the hospital and monitored during hospitalization over a 15-month period, from May 2017 to August 2018. The sample was recruited by simple random sampling from the patient list. The medical departments admit approximately 900 patients per year. Using a confidence level of 95% and a margin of error of 5%, the sample size is calculated to be at least 270.

Inclusion and exclusion criteria

We included patients over 18 years of age who had completed a lipid panel including total cholesterol, triglycerides, and HDL-c. Pregnant women and those with liver or kidney disease were excluded because they were more likely to have secondary dyslipidemia. Patients who refused to participate were also excluded.

Studied variables and definitions

We collected sociodemographic characteristics, anthropometric data, history of hypertension and diabetes mellitus, use of lipid-lowering medications, and lipid panel results. Patients were classified into four groups according to their occupation: primary sector (agriculture, fishing, mining), secondary sector (construction, building, industry, bakery, confectionery), tertiary sector (commerce, banking, insurance, transportation), and unemployed.

Total cholesterol, triglycerides, and HDL-c are measured directly in the blood. The Friedewald formula was used to calculate LDL-c. The types of dyslipidemia were pure hypercholesterolemia, mixed hyperlipidemia, hypertriglyceridemia, hypo-HDL-c, and hyper-LDL-c. Pure hypercholesterolemia was defined as total cholesterol greater than 5.67 mmol/L and triglycerides less than 2.28 mmol/L. Mixed hyperlipidemia was defined as total cholesterol greater than 5.67 mmol/L and triglycerides greater than 2.28 mmol/L. Hypertriglyceridemia was defined as total cholesterol less than 5.67 mmol/L and triglycerides greater than 2.28 mmol/L. Hypo-HDL-c was characterized by HDL-c less than 1.16 mmol/L in men and 1.42 mmol/L in women [14].

Body mass index (BMI) was calculated from weight and height. Normal reference values ranged from 17 to 24 kg/m^2^. Overweight was defined as BMI greater than 25 kg/m^2^, obesity as BMI greater than 30 kg/m^2^, and morbid obesity as greater than 40 kg/m^2^. Abdominal obesity was assessed by waist circumference, defined as greater than 102 cm for men and 88 cm for women [15].

Statistical analysis

Quantitative variables were expressed as means and/or medians. Qualitative variables were presented as frequencies and percentages. The chi-squared test was used for univariate analysis to determine any factors associated with dyslipidemia. P-values less than 0.05 were considered statistically significant. Analyses were performed using IBM SPSS Statistics for Windows, Version 22 (Released 2013; IBM Corp., Armonk, New York, United States).

Ethical considerations

The study was approved by the Research Ethics Committee of our institution, the University of Mahajanga. Informed consent was obtained from patients before data collection. Refusal to participate did not affect medical management and care. Patient anonymity was maintained.

## Results

A total of 384 patients were included. Table 1 shows the demographic and clinical characteristics of the patients. The mean age of the patients was 55.96 years (± 13.75), with extremes of 18 and 96 years. The sex ratio was 0.8, with 166 men and 218 women. The mean BMI was 24.75 kg/m^2^ (±5.43), with extremes of 11.11 and 58 kg/m^2^. The mean waist circumference was 90.78 cm (±14) in men and 93.55 cm (±15.14) in women. Thirty-nine patients (10.16%) were usually treated with lipid-lowering agents. Table 2 shows the mean values of the lipid panel results.

**Table 1:** Patient demographics and clinical characteristics

Demographic and clinical characteristics		Number (n = 384)	Percentage
Gender	Women	218	56.77
	Men	166	43.23
Age group (years)	18-35	32	8.33
	35-50	83	21.61
	50-65	160	41.67
	≥65	109	28.39
Occupation	Primary sector	31	8.07
	Secondary sector	14	3.65
	Tertiary sector	336	87.50
	Unemployed	3	0.78
Educational level	None	12	3.13
	Primary	104	27.08
	Secondary	206	53.65
	University	62	16.15
Hypertension	Yes	310	80.73
	No	74	19.27
Diabetes mellitus	Yes	63	16.41
	No	321	83.59
Lipid-lowering drug	Yes	39	10.16
	No	345	89.84
Body mass index	Healthy weight	208	54.17
	Overweight	120	31.25
	Obesity	51	13.28
	Morbid obesity	5	1.30
Abdominal obesity	Yes	174	45.31
	No	210	54.69

**Table 2:** Means of lipid panel results HDL-c: high-density lipoprotein cholesterol; LDL-c: low-density lipoprotein cholesterol

Lipid types	Women (n = 218)	Men (n = 166)	Total (n = 384)
Total cholesterol (mmol/L)	5.02 (±1.38)	4.59 (±1.29)	4.83 (±1.36)
HDL-c (mmol/L)	1.1 (±0.52)	1.08 (±0.46)	1.09 (±0.5)
LDL-c (mmol/L)	3.35 (±1.26)	2.96 (±1.17)	3.18 (±1.24)
Triglycerides (mmol/L)	1.28 (±0.91)	1.19 (±0.54)	1.24 (±0.77)

**Figure 1:**
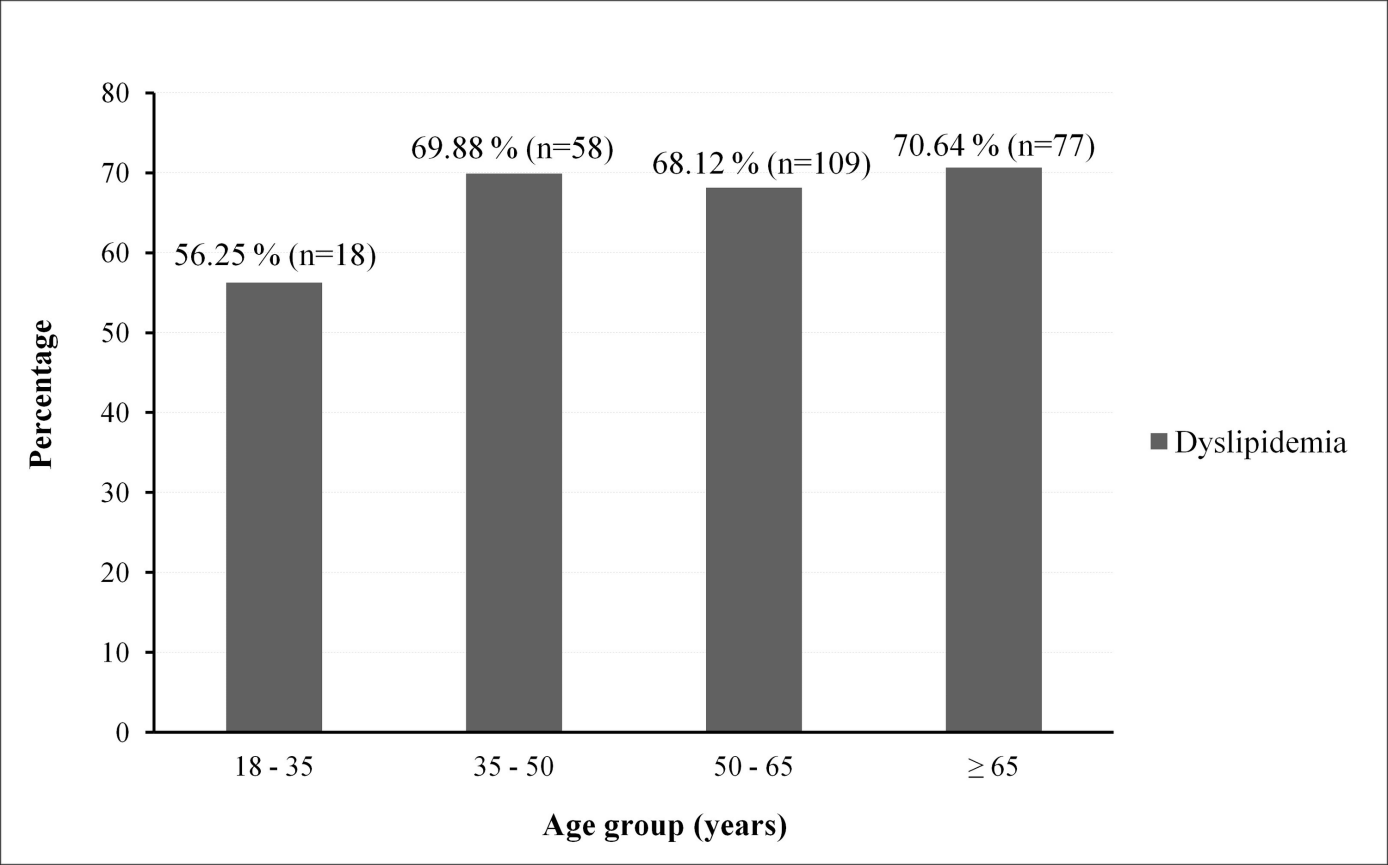
Prevalence of dyslipidemia by age

**Figure 2:**
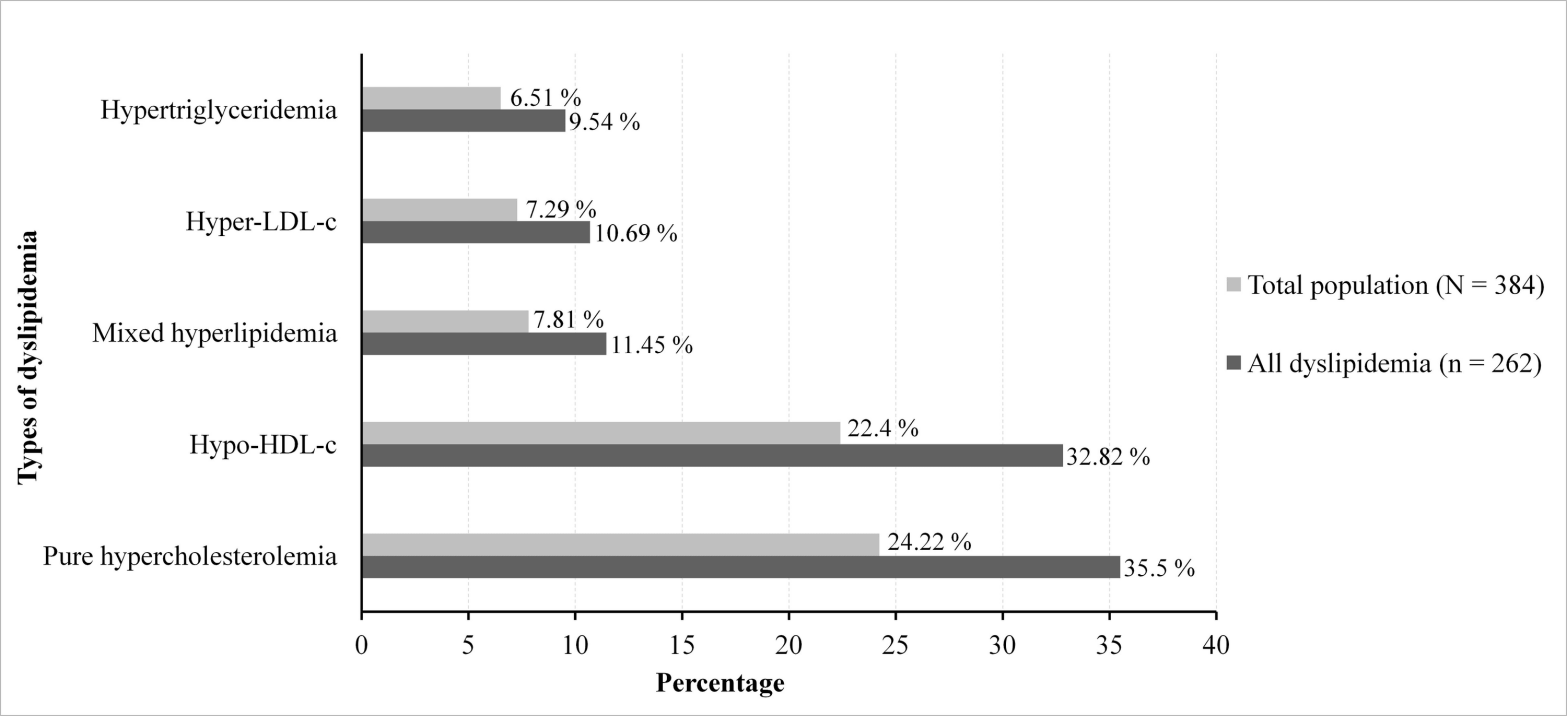
Types of dyslipidemia HDL-c: high-density lipoprotein cholesterol; LDL-c: low-density lipoprotein cholesterol

**Figure 3:**
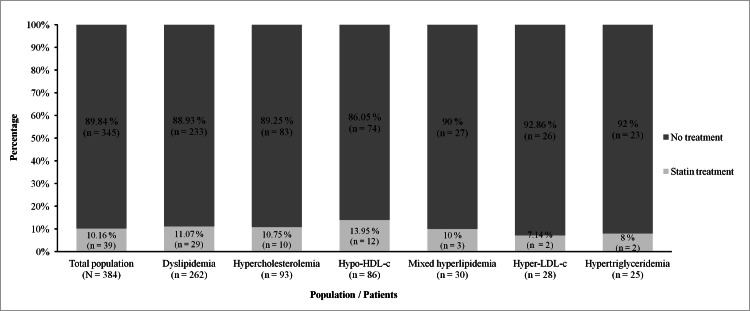
Distribution of dyslipidemia by statin treatment HDL-c: high-density lipoprotein cholesterol; LDL-c: low-density lipoprotein cholesterol

Of the 384 patients, 262 had an abnormal lipid profile, giving a prevalence of 68.23%. These patients had a mean age of 56.47 years (±13.47) and a sex ratio of 0.6, with 102 men and 160 women. The mean age was 58.45 (±12.69) years for men and 55.2 (±13.83) years for women. The prevalence of dyslipidemia was 61.45% in men and 73.39% in women. Figure 1 shows the prevalence according to age. Figure 2 shows the different types of dyslipidemia. Figure 3 shows the proportion of patients treated with lipid-lowering agents.

The frequencies of dyslipidemia according to gender, age, and comorbidities are shown in Table 3. Female gender (p = 0.013), diabetes mellitus (p = 0.007), and morbid obesity (p = 0.036) were associated with dyslipidemia (Table 4).

**Table 3:** Distribution of dyslipidemia by gender, age, and cardiovascular comorbidities HDL-c: high-density lipoprotein cholesterol; LDL-c: low-density lipoprotein cholesterol

Variables	Hypercholesterolemia n (%)	Hypo-HDL-c n (%)	Mixed hyperlipidemia n (%)	Hyper-LDL-c n (%)	Hypertriglyceridemia n (%)	Normal lipid n (%)
Gender						
Women (n = 218)	58 (26.61)	49 (22.48)	20 (9.17)	22 (10.09)	11 (5.05)	58 (26.61)
Men (n = 166)	35 (21.08)	37 (22.29)	10 (6.02)	6 (3.61)	14 (8.43)	64 (38.55)
Age group (years)						
18-35 (n = 32)	3 (9.38)	10 (31.25)	1 (3.13)	2 (6.25)	2 (6.25)	14 (43.75)
35-50 (n = 83)	29 (34.94)	13 (15.66)	2 (2.41)	6 (7.23)	8 (9.64)	25 (30.12)
50-65 (n = 160)	36 (22.5)	36 (22.5)	17 (10.63)	10 (6.25)	10 (6.25)	51 (31.88)
≥65 (n = 109)	25 (22.94)	27 (24.77)	10 (9.17)	10 (9.17)	5 (4.59)	32 (29.36)
Hypertension						
Yes (n=310)	84 (27.1)	54 (17.42)	26 (8.39)	26 (8.39)	20 (6.45)	100 (32.25)
No (n=74)	9 (12.16)	32 (43.24)	4 (5.41)	2 (2.7)	5 (6.76)	22 (29.73)
Diabetes mellitus						
Yes (n=63)	12 (19.05)	17 (26.98)	13 (20.63)	4 (6.35)	6 (9.52)	11 (17.46)
No (n=321)	81 (25.23)	69 (21.5)	17 (5.3)	24 (7.48)	19 (5.92)	111 (34.58)
Body mass index						
Healthy weight (n = 208)	48 (23.08)	56 (26.92)	12 (5.77)	10 (4.81)	10 (4.81)	72 (34.62)
Overweight (n = 120)	32 (26.67)	20 (16.67)	11 (9.17)	15 (12.5)	11 (9.17)	31 (25.83)
Obesity (n = 51)	13 (25.49)	10 (19.61)	7 (13.73)	3 (5.88)	3 (5.88)	15 (29.41)
Morbid obesity (n = 5)	0	0	0	0	1 (20)	4 (80)
Abdominal obesity						
Yes (n = 174)	48 (27.59)	30 (17.24)	16 (9.2)	20 (11.49)	11 (6.32)	49 (28.16)
Non (n = 210)	45 (21.43)	56 (26.67)	14 (6.67)	8 (3.81)	14 (6.67)	73 (34.76)

**Table 4:** Factors associated with dyslipidemia ref: reference

Demographic and clinical characteristics		Dyslipidemia		Total (n = 384)	p-value
		Yes n (%)	No n (%)		
Gender	Women	160 (73.39)	58 (26.61)	218	0.013
	Men	102 (61.45)	64 (38.55)	166	
Age group (years)	18-35	18 (56.25)	14 (43.75)	32	ref
	35-50	58 (69.88)	25 (30.12)	83	0.168
	50-65	109 (68.12)	51 (31.88)	160	0.196
	≥65	77 (70.64)	32 (29.36)	109	0.128
Occupation	Primary sector	18 (58.06)	13 (41.94)	31	ref
	Secondary sector	10 (71.43)	5 (28.57)	14	0.579
	Tertiary sector	231 (68.75)	105 (31.25)	336	0.223
	Unemployed	3 (100)	0	3	0.159
Education level	None	7 (58.33)	5 (41.67)	12	0.458
	Primary	75 (72.12)	29 (27.88)	104	0.705
	Secondary	137 (66.5)	69 (33.5)	206	0.675
	University	43 (69.35)	19 (30.65)	62	ref
Hypertension	Yes	210 (67.74)	100 (32.26)	310	0.675
	No	52 (70.27)	22 (29.73)	74	
Diabetes mellitus	Yes	52 (82.54)	11 (17.46)	63	0.007
	No	210 (65.42)	111 (34.58)	321	
Lipid-lowering drug	Yes	29 (74.36)	10 (25.64)	39	0.386
	No	233 (67.54)	112 (32.46)	345	
Body mass index	Healthy weight	136 (65.38)	72 (34.62)	208	ref
	Overweight	89 (74.17)	31 (25.83)	120	0.099
	Obesity	36 (70.59)	15 (29.41)	51	0.481
	Morbid obesity	1 (20)	4 (80)	5	0.036
Abdominal obesity	Yes	125 (71.84)	49 (28.16)	174	0.167
	No	137 (65.24)	73 (34.76)	210	

## Discussion

Dyslipidemia is multifactorial, involving genetic mechanisms and/or secondary causes. Primary dyslipidemia is an autosomal dominant disorder caused by mutations in the LDL-c receptor gene. This leads to a decrease in plasma LDL-c clearance and an increase in total cholesterol levels [16]. Secondary dyslipidemia is caused by diseases such as diabetes, chronic kidney disease, nephrotic syndrome, cirrhosis, and HIV infection. In this study, we excluded patients with liver or kidney disease. However, whatever the mechanism, dyslipidemia is a major risk factor for CVD [4].

Overall prevalence of dyslipidemia

In the Malagasy literature, Rabenjarison et al. (2016) [17] reported a prevalence of 46.17% in a 24-month study of 157 patients in Antananarivo, the capital of Madagascar. The prevalence of dyslipidemia in our study (68.23%) is higher than in the previous study. This can be explained by our increasingly unhealthy eating habits [17].

In the African literature, Thiombiano et al. in 2016 [18] reported a prevalence of 61.3% among individuals residing in Guéoul (Senegal). In a meta-analysis of data from 2022 [19], the prevalence in the East African region was 60.7%, which is similar to ours. In 2022, Masilela et al. [20] conducted a study in primary care centers in South Africa, and the results showed a higher prevalence (76.71%).

In the Asian literature, Xing et al. [21] reported a prevalence of 35.8% in Liaoning Province (China) in 2020. In 2021, Gao et al. [22] reported a prevalence of 48.27% in Shenmu City (China). Our prevalence remains higher than these data.

In Europe, nearly 20% of patients aged 50 years or older with a cardiovascular risk factor have dyslipidemia, according to data reported in 2017 from the European Study on Cardiovascular Risk Prevention and Management in Usual Daily Practice (EURIKA) study [23]. In the UK, the prevalence of dyslipidemia increased from 13.5% in 2009 to 23.5% in 2019, according to Bilitou et al. [24]. Our prevalence is higher than these findings.

Several observations reflect the importance of socioeconomic status, ethnic background, and genetic factors in the prevalence of dyslipidemia [1,25]. It is therefore essential to recognize cultural differences in health care to improve outcomes in the management of CVD [26].

Prevalence of dyslipidemia by gender and age

The prevalence of dyslipidemia was higher in women (73.39%). Similar results were observed in Senegal in 2016 (65.4%) [18], in South Africa in 2022 (75.79%) [20], and in the EURIKA study data (54.87%) [23]. This could be due to the frequent accumulation of fat in women and the decrease in hormonal protection after menopause [27]. On the other hand, some authors have reported results with a high prevalence in men [17,21,22,28].

According to age, the prevalence of dyslipidemia was highest in patients older than 65 years (70.64%) (Figure 1). In the previous Malagasy study, patients over 60 years of age were most affected (42.02%) [17]. Age is an important cardiovascular risk factor. Age is responsible for disturbances in lipoprotein metabolism characterized by changes in hepatic sinusoidal endothelium, peroxisome activity, and increased insulin resistance induced by free fatty acids [28,29].

Patient lipid profile

In our study, pure hypercholesterolemia (24.22%) and hypo-HDL-c (22.4%) were the most common. Hypertriglyceridemia (6.51%) was less frequent. In 2005, the French MONICA study [30] reported a high prevalence of pure hypercholesterolemia (30%) and hypo-HDL-c (12%) and a low prevalence of mixed hyperlipidemia (5%) and hypertriglyceridemia (4%). Our results are consistent with this Multinational MONItoring of Trends and Determinants in CArdiovascular Disease (MONICA) study and other studies in the literature regarding the predominance of pure hypercholesterolemia and hypo-HDL-c [31,32]. Furthermore, Rabenjarison et al. [17] reported a predominance of hypercholesterolemia (54.77%) and hypertriglyceridemia (35.03%), similar to data observed in South Africa (Masilela et al. 2022) [20], China (Xing et al. 2020; Gao et al. 2021) [21,22] and Bangladesh (Ali et al. 2023) [28]. The variation in results could be explained by the different nature and size of the populations and, most importantly, the context of lipid-lowering drug treatment prior to lipid blood testing.

Thirty-nine patients were receiving lipid-lowering medication, representing 10.16% of participants and 11.07% of patients with dyslipidemia. In the study by Masilela et al. [20] in South Africa, only 16.78% of participants were treated with lipid-lowering drugs. The proportion of treated patients remains low in low-income countries, contributing to the increase in cardiovascular morbidity and mortality and explaining the higher prevalence of dyslipidemia [33]. In contrast, data from the USA showed a 79% increase in statin use between 2002 and 2013 [34]. In European countries, the proportion of treated patients was well over 30% [23,30].

Factors associated with dyslipidemia

Female gender (p = 0.013) was associated with dyslipidemia. Although several studies have found opposite results, the EURIKA study showed that female gender was positively associated with hypo-HDL-c but negatively associated with hypertriglyceridemia [23]. In Tanzania, a study of HIV-infected patients over 15 years of age showed that female gender was a predictive factor for dyslipidemia [35]. The association with the female gender may be explained by the menopausal transition and loss of estrogen in older women, which exacerbates metabolic dysfunction [27].

Diabetes mellitus was associated with dyslipidemia (p = 0.007). The prevalence of dyslipidemia in diabetic patients was 82.54%, with a high frequency of hypo-HDL-c (26.98%). These results were also found in other studies with a predominance of hypo-HDL-c [20]. In diabetes, insulin resistance, hyperglycemia, and hyperinsulinemia lead to activation of cholesteryl ester transfer protein, which is responsible for increased HDL-c catabolism [36].

Morbid obesity was associated with dyslipidemia (p = 0.036), with hypertriglyceridemia being the most common. Dyslipidemia in obesity is mainly characterized by elevated triglycerides, decreased HDL-c levels, and normal or slightly elevated LDL-c levels. Obesity-related lipid abnormalities often result from decreased clearance of triglyceride-rich lipoproteins [37]. They may also be associated with increased production of apoprotein B, resulting in elevated LDL-c. Weight management is therefore essential in the prevention of CVD.

Study strengths and limitations

This study has limitations. Dyslipidemia was defined by biological criteria, although some patients were already treated with a lipid-lowering agent. This may have underestimated the prevalence of dyslipidemia. The influence of social factors on our results is not very clear, which raises questions about the generalizability of these results to the population as a whole. When comparing our results with the literature, it is important to note that we cannot be sure that the populations are truly comparable. However, the study reported the prevalence of dyslipidemia specifically in Malagasy patients. The number of participants was higher than in the previous study. The proportion of patients receiving lipid-lowering treatment was low. These results reflect the challenge of managing cardiovascular risk factors.

## Conclusions

The prevalence of dyslipidemia varies according to socioeconomic status, ethnic background, and genetic factors. In our study, the prevalence of dyslipidemia was high. The frequency of patients treated with lipid-lowering agents was low, similar to observations in other low-income countries. Female gender, diabetes mellitus, and obesity were the factors associated with dyslipidemia. Interventions for CVD need to be culturally appropriate to increase patient engagement and adherence.
